# Public health communication in Canada during the COVID-19 pandemic

**DOI:** 10.17269/s41997-022-00702-z

**Published:** 2022-11-03

**Authors:** Maya Lowe, Shawn H. E. Harmon, Ksenia Kholina, Rachel Parker, Janice E. Graham

**Affiliations:** 1 Department of Pediatrics (Infectious Diseases), Faculty of Medicine, Dalhousie University, Halifax, NS Canada; 2 Technoscience & Regulation Research Unit, Faculty of Medicine, Dalhousie University, Halifax, NS Canada

**Keywords:** Health communication, COVID-19, Pandemic response, Governance, Transparency, Community engagement, Communication en santé, COVID-19, réponse à une pandémie, gouvernance, transparence, engagement communautaire

## Abstract

**Objectives:**

Communication is central to the implementation and effectiveness of public health measures. Informed by theories of good governance, COVID-19 pandemic public health messaging in 3 Canadian provinces is assessed for its potential to encourage or undermine public trust and adherence.

**Methods:**

This study employed a mixed-methods constant comparative approach to triangulate epidemiological COVID-19 data and qualitative data from news releases, press briefings, and key informant interviews. Communications were analyzed from January 2020 to October 2021 in Nova Scotia, Ontario, and Alberta. Interview data came from 34 semi-structured key informant interviews with public health actors across Canada. Team-based coding and thematic analysis were conducted to analyze communications and interview transcripts.

**Results:**

Four main themes emerged as integral to good communication: transparency, promptness, clarity, and engagement of diverse communities. Our data indicate that a lack of transparency surrounding evidence and public health decision-making, delays in public health communications, unclear and inconsistent terminology and activities within and across jurisdictions, and communications that did not consider or engage diverse communities’ perspectives may have decreased the effectiveness of public health communications and adherence to public health measures throughout the COVID-19 pandemic.

**Conclusion:**

This study suggests that increased federal guidance with wider jurisdictional collaboration backed by transparent evidence could improve the effectiveness of communication practices by instilling public trust and adherence with public health measures. Effective communication should be transparent, supported by reliable evidence, prompt, clear, consistent, and sensitive to diverse values. Improved communication training, established engagement infrastructure, and increased collaborations and diversity of decision-makers and communicators are recommended.

## Résumé

**Objectifs:**

La communication est centrale à l’implantation et au succès des mesures de santé publique. À partir des théories de bonne gouvernance, les messages de santé publique sur la COVID-19 de trois provinces canadiennes ont été analysés afin d’évaluer si ces messages renforçaient ou diminuaient la confiance et l’adhésion de la population.

**Méthodes:**

Cette étude utilise une approche comparative constante et des méthodes mixtes pour trianguler des données épidémiologiques sur la COVID-19 avec des données qualitatives provenant de communiqués de presse, de conférences de presse et d’entretiens avec des informateurs clés. Les communications ont été analysées entre janvier 2020 et octobre 2021 en Nouvelle-Écosse, Ontario et Alberta. Les données proviennent également de 34 entretiens semi-dirigés avec des acteurs de santé publique à travers le Canada. Une codification en équipe et une analyse de contenu thématique ont été réalisées pour analyser les communications et les verbatim des entretiens.

**Résultats:**

Quatre thèmes principaux sont apparus comme faisant partie intégrante d’une bonne communication : la transparence, la rapidité, la clarté et l’engagement des diverses communautés. Nos données indiquent qu’un manque de transparence en ce qui a trait aux données probantes et aux processus de prise de décision en santé publique, que les retards dans les stratégies de communication de santé publique, qu’une terminologie et des activités incohérentes et inconsistantes dans les provinces et entre elles, ainsi que des communications qui ne tenaient pas compte des points de vue ou qui n’incluaient pas les diverses communautés, ont pu réduire l’efficacité des communications et l’adhésion aux mesures de santé publique tout au long de la pandémie de COVID-19.

**Conclusion:**

Cette étude suggère que des lignes directrices nationales et une collaboration accrue entre les provinces et les territoires soutenues par des données probantes transparentes pourraient améliorer l’efficacité des communications en suscitant la confiance du public et l’adhésion aux mesures de santé publique. Une communication efficace doit être transparente, appuyée sur des données probantes fiables, rapide, claire, cohérente et sensible aux différentes valeurs. Il est recommandé d’améliorer la formation en communication, d’établir une infrastructure pour accroître l’engagement, et de soutenir la collaboration et la diversité des décideurs et des communicateurs.

## Introduction

As the COVID-19 pandemic unfolded across Canada in 2020, public health officials gained prominence, even centre stage. Amid scientific uncertainty, initial communication focused on public health measures (PHMs) undertaken by ordinary citizens (e.g., physical distancing, reduced social contact, masking), then turned to encouraging vaccination as vaccines became available. While Canada outperformed most G10 countries in terms of fewer COVID-related infections and deaths, and higher vaccination uptake (Razak et al., 2022), its management of the pandemic was lacking (Yu et al., 2021). Shortcomings in governance and insufficient attention to public interest may have contributed to failures in pandemic response (Perry et al., 2014).

As a continuing process through which diverse and often conflicting interests are accommodated to achieve social ends, key features of ‘good governance’ include a process based on coordination, not control; participation by public and private sectors/actors; and ongoing engagement (Commission on Global Governance, 1995). The more aligned that policy and decision-making practices are to this, the better that countries tend to perform during emergencies (Nabin et al., 2021). The essential characteristics of good governance are widely considered to be legitimacy, transparency, accountability, responsiveness, effectiveness, and rule of law (Keping, 2018; Addink, 2019; Nabin et al., 2021). Central to the characteristics of good governance is ‘good communication’ (Addink, 2019) and the absence of these conditions can undermine trust in decision-makers and their message when such trust is necessary for dialogical and participative ‘good governance’ and successful crisis management (Adhani et al., 2022; Khosravi, 2020; Lee & Li, 2021; Salmon et al., 2021). Trust has profound implications for health outcomes (Lee & Li, 2021). Where trust is precarious, undermined, or absent, people may be unwilling to hear critical messages and align their behaviour to PHMs communicated (Hyland-Wood et al., 2021; Khosravi, 2020; Ryan et al., 2019). The qualities of good communication (i.e., communication that supports regulatory legitimacy and advances good governance) include transparency, promptness, clarity, and engagement (Jones & Graham, 2009; Hyland-Wood et al., 2021):

- **Transparency**: Communication must convey up-to-date knowledge based on the ‘best’ open and reliable evidence, and disclose decision-making processes and interests of information decision-makers so that actors can form appropriate opinions about risk, expectations, and behaviour.
- **Promptness**: Communication must be timely to encourage actors to collectively work toward stated objectives clearly conveyed.
- **Clarity**: Communication must be clear, comprehensible, and coordinated within and across actors and media to minimize confusion.
- **Engagement**: Communication must be capable of building relationships, bringing people together in pursuit of shared objectives—rather than dividing or ‘othering’ them—an outcome achieved by engaging with publics and taking into account diverse identities, values, and experiences.

This pan-Canadian study of law as an enabler of and barrier to public health examines public health messaging in Nova Scotia, Ontario, and Alberta relating to COVID-19 PHMs, considering their potential to encourage or undermine trust by how well they reflect the above characteristics of good communication.

## Methods

In Canada, provinces have primary responsibility for healthcare delivery. Absent a federal declaration of national emergency in response to COVID-19, the provinces carried the ball in designing PHMs within their borders. We selected three provinces—Nova Scotia, Ontario, and Alberta—for this comparative analysis based on their geographical separation, mix of population density, socio-cultural demographics, economic conditions, political climate, and pandemic experiences. We applied a mixed-methods constant comparative approach (Glaser, 1965) to triangulate epidemiological data (COVID-19 case counts, mortality, vaccine uptake) and qualitative data (news releases, press briefings, key informant interviews). Ethical approval was obtained from the IWK Health Centre Research Ethics Board (#1025970).

Epidemiological data were collected from Public Health Infobase (PHAC, 2020) for the period 31 January 2020–5 October 2021. For the same period, key PHM updates were extracted from respective provincial websites (Appendix Table 1).

Qualitative data were collected from multiple sources. A baseline of government interventions from March 2020 to October 2021 was developed based on news releases and a publicly available government action timetable (McCarthy Tetrault, 2022). Once key dates were identified within our target timeframe, we examined temporally proximate public health press briefings, supplemented by popular media articles reporting on the press briefings sourced through Google. Key informant interviews were conducted from September to December 2021 via Zoom. Participants were recruited from across Canada using a combination of purposive and snowball sampling of public health experts. Data saturation was achieved insofar as final interviews resulted in response repetition and the absence of new issues raised. Transcripts were produced and edited using Otter.

Data were coded and analyzed in NVivo. A structured three-stage team-based coding approach was employed (Giesen & Roeser, 2020) involving initial coding (usually by the lead interviewer), joint coding (by first coder and reviewer), and consistency coding (by reviewer). Using a thematic analysis approach, codes were organized into categories under emerging themes (Braun & Clarke, 2006). All data were triangulated using constant comparative analysis (Glaser, 1965) that evaluated emerging themes across data gathered (Fram, 2013) until saturation. Participant responses were analyzed to discern their agreement with the conditions of good communication. Excerpts from interviews are used below where they reflect on one or more conditions, and where they are indicative of multiple participant opinions.

While federal authorities were also communicating with the public, our emphasis was on provincial interventions, which were not uniformly or collaboratively introduced. Therefore, recommendations offered in relation to federal communication or federal leadership pertaining to government or emergency communication are driven by our findings on the quality of provincial communication in the select jurisdictions, as well as expert interviews.

## Results

**Fig. 1:**
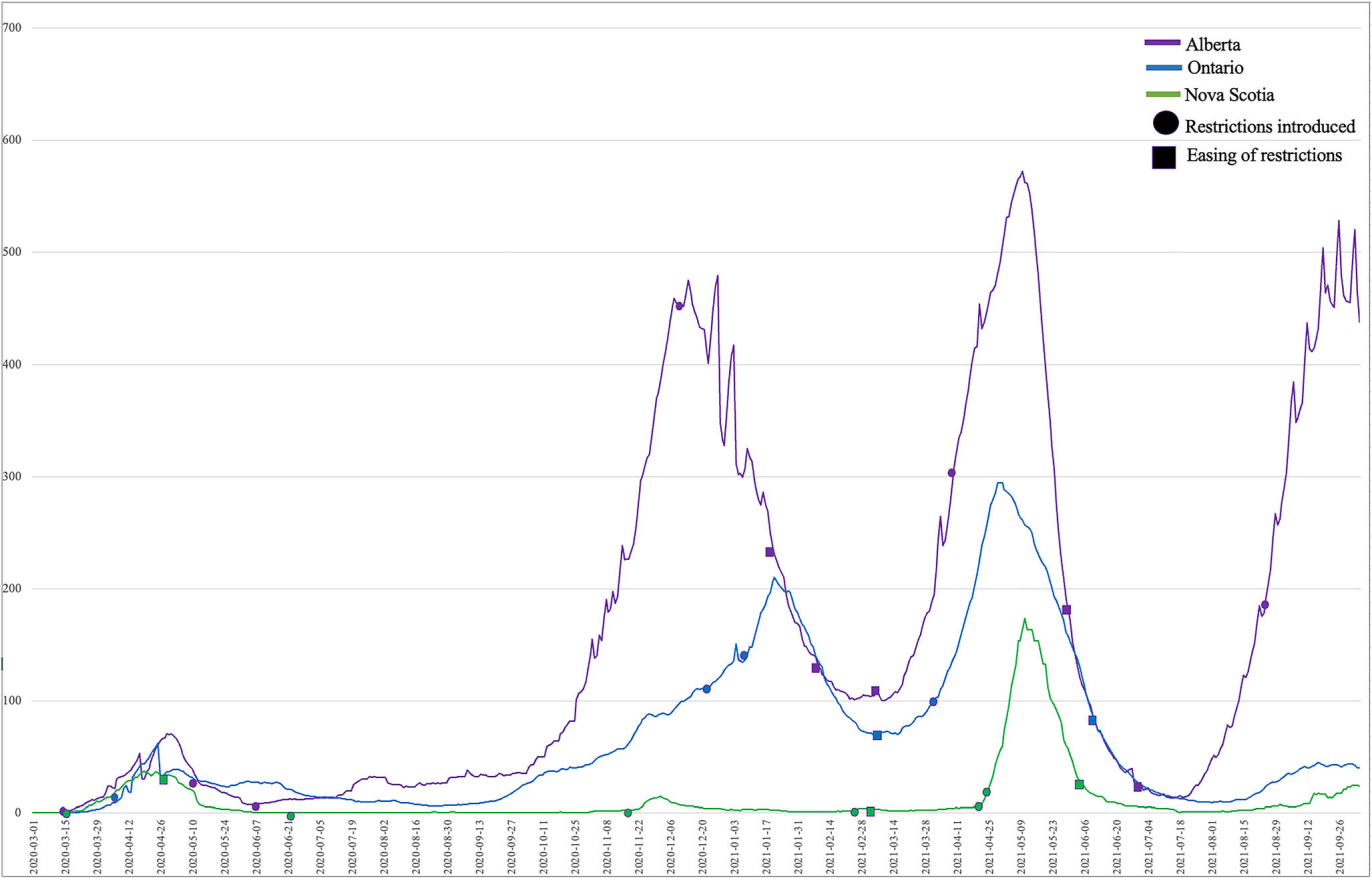
COVID-19 cases (per 100,000) and key PHM communications by province

In total, 65 news releases were analyzed from Alberta, 56 from Ontario, and 166 from Nova Scotia (Appendix Table 1). We identified 36 press briefings from Alberta, 17 from Ontario, and 7 from Nova Scotia that met our condition of serving as a live government announcement of PHMs, often accompanied by a media question/answer session (Appendix Table 2). Figure 1 illustrates the proportion of active COVID-19 cases (per 100,000) during the study period, underlining a sample of inconsistent and sometimes arbitrary enactments of PHMs.

A total of 34 interviews were conducted with key informants from one or more of 11 jurisdictions (global, federal, Alberta, British Columbia, New Brunswick, Northwest Territories, Nova Scotia, Ontario, Prince Edward Island, Saskatchewan, Quebec). Participants represent one or more of four cohorts: public health officials, frontline healthcare workers, health scholars (social, epidemiological, policy, and clinical researchers), and healthcare worker union leaders.

Constant comparison of critical incidents across data sources point to four conditions that may improve public health communication: transparency, promptness, clarity, and engagement.

### Transparency

Transparency is a complex condition of communication that involves evidence and disclosure of interests and processes. In a pandemic where information, misinformation, disinformation, and conspiracy theories circulate, scientific evidence is instrumental in countering skepticism, justifying PHMs, and fostering public trust in those PHMs. Throughout the pandemic, public health officials attempted to justify actions with information to gain public trust and compliance with PHMs, but efforts were uneven at best. During autumn 2020 in Alberta, Premier Jason Kenney cited evidence to support his decision to keep restaurants open:

We’re following data, we’re following evidence. People who are hounding us to shut down restaurants—Why? Because they want us to, quote, do something when our data indicates that 0.7% of identifiable transmission has occurred in restaurants and similar businesses? (CPAC, 2020f)

During summer 2021, Chief Medical Officer of Health (CMOH) Dr. Kieran Moore (ON) addressed fertility concerns related to COVID-19 vaccines, saying that officials were paying ‘close attention’ to a study following 40,000 pregnant women in Ontario, all of whom had been vaccinated safely without complications (CPAC, 2021f).

Participants spoke about challenges addressing false information. FL5, an infectious disease paediatrician, stated:

People […] like to ‘do their research.’ And that can be a problem because they go to deep corners of the internet, and they get wrong information […]. And so, it’s important to emphasize strategies that provide them with information that is correct […] so that, they can make the right decisions. And in providing them with correct information, coming up with different strategies but also connecting them with […] different pathways of information.

Directing hesitant individuals to reliable, trustworthy sources to enable informed choices regarding immunization is particularly critical as PH8, a provincial health officer, acknowledged:

Anti-vaxxers are using fear tactics and they don’t have to say things that are true. But they can say things that are scary or completely biased. And so, it’s not a fair game. […] I think we need to beef up our capacity to deal with things like that. So, that might be a reform in the sense of paying more attention to it, supporting it more using evidence in terms of what works, from a public perspective, a broad perspective, in terms of messaging, and how do you modify people’s understanding […] to align […] their beliefs and their thinking […] with accurate information. And I think that’s […] one of the big challenges.

Sharing reliable, evidence-based information through diverse media can help reach multiple communities and dispel inaccurate information circulating online. This requires awareness of where misinformation comes from, and emotions, such as fear, associated with immunization decisions. Reliable information must be conveyed clearly, and in ways that are understood. FL8, an Alberta healthcare worker, warned that purely scientific data without integrating social factors, including health literacy, can harm public understanding:

Doctors and public health professionals are up here all the time, talking about efficacy. I had a client talk to me at one of the vaccine clinics, and she said, “I’m so glad to hear the vaccine has an 80% efficacy rate. It’s just such a shame that 20% of the people will die.” Like, that was the way that the information was being read […]. Efficacy [is] a tough mathematical idea to understand.

Vaccine communication is most persuasive when it is reliable, evidence-based, and clear about risk, and avoids blame and stigma-generation (Bardosh et al., 2022; MacDonald et al., 2021). It was not uncommon for government communicators to shame those who were not vaccinated, a common refrain being that “this is a pandemic of the unvaccinated.” S4 recognized the harm in shaming:

We’re really not giving people who have not been vaccinated a chance to feel good about getting vaccinated. We’ve already moved, like, fully into the shame mode, right? And that’s going to be hard for some people to walk back if they’ve got any kind of pride. So, you can try to force them with vaccine passports. But you know, they’re going to be resistant. So, we haven’t gotten that piece right.

Transparency concerning what can be expected from rapidly emerging information is not often achieved. FL3, an Ontario physician, noted the disconnect between common beliefs about what vaccines are meant to protect against, and how the COVID-19 vaccines actually performed, describing how they protected from severe symptoms but not as well from getting infected, yet officials initially failed to convey this or other evidentiary gaps. Missing evidence relating to the different efficacy and safety profiles of viral vector and mRNA vaccines also contributed to confusion (Szklarski, 2021). Government communicators spoke of vaccine adverse events without specifying at-risk groups associated with demographics and predisposing conditions. Again, public perceptions turned negative. FL5 acknowledged the challenges posed, and the utility of federal guidance:

You need to control the release of information. You need a structured pathway through which information flows. The provinces should buy into that. It avoids mixed messages [and] confusion when decisions are made. […] I think the communication relating to the AstraZeneca vaccine and clots associated with that could have been handled differently. And that’s one example of decisions that are made regarding communication at a federal level versus at provincial levels.

FL6, a primary care physician in Alberta, stated:

They were like, “You should take it!” And then they were like, “No, you shouldn’t!” And they were like, “Well, if there’s enough COVID in your area, you should. And if you’re under this age, you should.” […] They were saying, “Yeah, there’s a risk of this happening with AstraZeneca. But for those of you in Calgary—at that point it was a total disaster—yeah, get AstraZeneca because your risks if you get COVID are far higher.” But that’s a confusing concept, I think, for a lot of people. And it felt very tied to emotions and […] so maybe a little bit less flip-flopping would have been helpful.

Ultimately, the handling of vaccine-related concerns lacked transparency, reliable evidence, and sufficient engagement with the public. Enhanced federal guidance or leadership on this matter might have eased confusion among and within jurisdictions and fostered public trust by providing a consolidated evidence-based response. S4 suggested that underlining the impact of vaccines on death rates, being clearer about the need for future boosters, and emphasizing that vaccination is one step people can take for individual and collective protection, would have been better.

CMOH Dr. Robert Strang (NS) announced that the benefits of COVID-19 vaccines ‘far outweigh’ the potential risk of myocarditis or pericarditis, and emphasized the need to make informed decisions, although he did not identify where trustworthy information could be found (CPAC, 2021i). CMOH Moore (ON) urged those who were hesitant to speak to trusted healthcare providers (CPAC, 2021h), a point emphasized by eight interviewees, including S4:

Our Chief Medical Officers of Health, […] they’ve been doing a really hard job, [but] people would rather talk to their doctor [… or] the pharmacist about a vaccine. So, it really doesn’t help for someone on the TV to say, “AstraZeneca has side effects, but only for this narrow slice of the population.” It helps [...] to say, “This is a very good vaccine. It might not be the best vaccine for you. But talk to your doctor, and they’ll tell you which vaccine is the best one for you. One of the three or four—is going to be the best one for you.” I think that’s a much better way of communicating. […] Leave the conversation on particulars of the particular vaccines […] to people who are trusted.

As noted, officials occupy a competitive informational space that requires acknowledging and countering the information quagmire. CMOH Strang remarked, “No matter how many YouTube videos or conspiracy theories from so-called experts that you send to me, we will not agree. [...] Please, please, get vaccinated” (CPAC, 2021i). CMOH Moore, responding to concerns about vaccines on fertility, drew on evidence from consultations with the Society of Obstetricians & Gynecologists (CPAC, 2021f). FL8 suggested that knowing the audience and tending to emotional aspects can be effective:

They need to have a better understanding of where misinformation comes from and why people believe it. I think that’s been a […] really unique challenge to this vaccination campaign […] To use a pre-COVID example, if you say to a family, “Vaccines don’t cause autism. We have studies.” That’s true. But why do they think that? And what other beliefs have led them to this place? You can’t just say, “That’s not true.” Because that’s not emotionally compelling. It’s not persuasive. […] In terms of public health messaging, I think there have been some emotionally compelling things about “We’re all going to get back to doing things we love.” […] And I think in some cases, that’s been effective.

Ultimately, to achieve transparency, communication should be evidence-based, reliable, and tailored through wide engagement with diverse communities.

### Promptness

Rapid communication was prioritized in COVID-19 press briefings across jurisdictions, as well as among participants. PH5, a public health officer with provincial and federal expertise, stated:

[T]here just needs to be a lot of emphasis on our regulatory bodies, on Public Health Canada, on National Advisory Committees, on more timely advisories, more timely recommendations, better communication, whatever they need to do […] It needs to come quicker, faster and clearer than it is now […] Going forward [...], that is a big, big issue. So, for any new vaccines for that matter, recommendations need to come sooner.

FL4, an Ontario physician specializing in refugee health, emphasized timely communication in countering misinformation and anti-vaccination rhetoric:

We were too slow off the mark. I think some of the anti-vaccine messages had already set in […] November, December of 2020. And for some people, it just became very entrenched […]. And I think that’s true to this day. […] A lot of people have moved, but for the ones in my practice, that 8%, they are now immovable. And I think we could have messaged earlier.

In the summer of 2020, CMOH Dr. Deena Hinshaw, facing a backlash, apologized after Alberta issued Order 33-2020 regarding COVID-19 guidelines for schools without a public statement:

I am very sorry for the anxiety and confusion that this Order has sparked. This timing was not meant to hide information. Ironically, it was meant to be transparent. The intent was to ensure school authorities knew about the order, which codifies the guidance that has been online for weeks, before the order came into effect today. I understand the concerns, especially as we move forward quickly, and the need to ensure that Albertans have accurate information (CPAC, 2020e).

This illustrates the critical nature of prompt/timely public health communication during a pandemic with rapidly changing conditions and guidelines, and is seen to be important for instilling trust in governmental decision-making.

### Clarity

If people are to have confidence in the information they are receiving, it needs to be clear. That demands consistency in relation to common conditions. All four cohorts of participants emphasized the importance of consistent communication and commonality of core ideas. S4, a public policy scholar, emphasized the relationship between consistent messaging, public confusion, and trust, highlighting the need for justifications of PHMs:

If we’re doing this again, we have to put way more responsibility on the politicians to articulate why they’re allowing things to happen and how that thing contributes or doesn’t contribute to the end goal. And they’ve got to define what that end goal is. It can’t simply be […] to maintain or to control the virus. That’s not the reason why a society exists, right? Or the reason why a government is there. The government is there to […] help produce other big goods that we can all benefit from.

Press briefings were strikingly inconsistent and incoherent, particularly concerning vaccine quality, symptoms, adverse events following immunization, mixed dosing, and the fundamental matter of describing PHMs meant to interrupt virus transmission. Terms included ‘shutdown’, ‘lockdown’, ‘circuit-breaker’, and ‘stay-at-home order’ with variable and unclear meanings and significance. Bol et al. (2021) define a ‘lockdown’ as a form of nationwide social confinement in which citizens are forced, rather than simply encouraged, to stay at home unless leaving for a ‘valid’ reason. A ‘shutdown’ refers to a more drastic form of lockdown (Cuoto Zuber, 2021). A ‘circuit breaker’ is a type of lockdown that has a set end-date rather than one determined by target case counts (Mohan, 2020). Inconsistency in the communication of these terms generated feelings of frustration, defeat, and exhaustion, which undermined trust in officials and induced some to create their own rules (Cuoto Zuber, 2021).

Nova Scotia implemented two circuit-breakers and one shutdown in early 2021. A circuit-breaker announced on February 23 was rescinded 1 week later with the justification by CMOH Strang that he would rather “under-promise and over-deliver” (CPAC, 2021b). While Premier Iain Rankin contended that safety is a priority, he acknowledged the negative impact of restrictions on businesses (CPAC, 2021b). After the rescission, active cases spiked. The next circuit-breaker was not implemented until after cases had already declined, and it transitioned to a province-wide shutdown in April 2021. The delay and discordance between case numbers and implementation of further restriction did not build public confidence.

Ontario enacted one province-wide shutdown and two stay-at-home orders from December 2020 to April 2021, without articulating their differences. Public confusion around behavioural guidelines was ongoing, with uncertainty compounded by public officials failing to comply with their own guidelines (Brown, 2020). Facing public frustration, Premier Doug Ford insisted that the guidelines “could not be clearer”. After the second stay-at-home order, his competence in communicating with Ontarians in a consistent manner, and ability to lead the province through the third wave, was questioned. Without explanation, he remained absent from subsequent COVID-19 briefings after he was accused of having “blood on his hands” and that there were “concerns for his moral authority to lead the province” at a press briefing (CPAC, 2021c).

Although Alberta employed restrictions similar to those in other jurisdictions, there were no declared shutdowns or lockdowns. In May 2021, Premier Kenney explained that Alberta ‘resisted pressure’ to implement lockdowns, taking a “balanced approach, following the evidence” because “governments must not impair peoples’ rights, or their livelihoods, unless it is absolutely necessary to save lives” (CPAC, 2021e). Reticence to name restrictions as lockdowns or shutdowns contributed to inconsistencies and ambiguity between provinces.

### Engaged

The impact of communication—particularly risk communication—is not generated by raw data alone, but by the emotional involvement and sense-making of recipients (i.e., by their experience, identity, and values) (Engdahl & Lidskog, 2014). Complex, multicultural liberal democracies like Canada are shaped by many, often competing values, some of which may be pitted against each other in specific contexts or discourses (Wu et al., 2021). For example, individualism—emphasizing autonomy, rights, and perceived risks/benefits to individuals—and communitarianism—emphasizing solidarity, responsibilities, and risks/benefits to the community—may both be important, but they are often not explicitly expressed or given equal weight.

Throughout the pandemic, values were suggested through political positioning, but they were rarely explicitly referenced, nor was recognition given to the range of communities’ values, so potentially competing values were hardly ever reconciled. Having said that, CMOH Strang did this overtly when he emphasized individual *responsibility* instead of individual *entitlements*:

Personal choice cannot be all you think about when it comes to COVID vaccines. I would ask you to focus on others and that you focus on the ‘we’ and not the ‘me.’ The choice to be vaccinated or not has implications for everyone around you (CPAC, 2021g).

In Ontario in April 2020, CMOH Dr. David Williams highlighted the importance of wearing masks, not in fear of others but to protect them (CPAC, 2020d). Alberta CMOH Hinshaw took a slightly different tack; in an effort to encourage compliance with government recommendations, she emphasized *individual agency* and personal empowerment. In early March 2020, she said, “We are all responsible for each other’s health,” and “We all have a responsibility to prevent the spread of this virus” (CPAC, 2020a). Later in March, she reiterated, “I want to stress overall that the future of this pandemic is in all of our hands. We have a say in how COVID-19 will impact our province” (CPAC, 2020b). Premier Kenny further underlined this notion by stating that the course of the pandemic will be decided by the choices individuals make (CPAC, 2020c). S2, an immunization expert, highlighted the utility of emphasizing responsibility within the context of infectious disease, saying that the consequences to family and others of not being immunized were not explained consistently or well. The right to refuse immunization if one has a medical contraindication can be properly circumscribed by the right of others not to be infected as a result.

Local engagement facilitates practices that reflect community values. This is a particularly pressing and demanding requirement in Canada, with such diverse populations and experiences of government. Note should be taken of the legacy of dispossession, marginalization, and genocide experienced by Indigenous Peoples, their troubled history with Canadian institutions, and Indigenous-specific racism that persists in healthcare, all of which have left many Indigenous Peoples suspicious of healthcare workers and hesitant to accept vaccines (Mosby & Swidrovich, 2021). Attention must also be paid to the messenger. PH11, a federal public health official, commented on the importance of the communicator to effectively speak to equity-seeking groups:

There’s been colonization for Indigenous People. And there’s been experimentation […] without consent. So, there’s a lot of skepticism and distrust. [They wonder], “Hmm, is this another experiment?” Only, you know, when […] one involves the leaders of the community—the Elders in the Indigenous community context, or Indigenous doctors, nurses and other champions—they explain, “No, this is not that. COVID is a real danger. This is […] not someone trying to put microchips into our arms.” […] But that […] message would be much better received, if it’s coming [...] from the same community, leaders in the community. And that goes for other racialized marginalized groups as well.

FL5 confirmed the communicator’s important role:

I think it’s important to have individual people who are making decisions with respect to the vaccine rolling out, and making decisions with respect to communication, really look like the Canadian population. It speaks to diversity. And that includes ensuring that there’s adequate representation of certain groups like the Indigenous population, Black communities, other communities. It requires ensuring that there’s diversity in the people making decisions and in people who are doing the communication.

These excerpts highlight the importance of ongoing and trustworthy deliberation and engagement with diverse communities/perspectives, and of facilitating solutions through community communicators.

This value variance across Canada calls for more robust and refined ways to enhance communication through processes that engage, mobilize, and speak more powerfully to communities. Communication must be approached as a *collaborative practice* grounded in communities that permits local values, knowledge, experience, and needs to inform not only the nature of messaging but also the interventions expressed. Government communicators often failed to achieve compassionate communication that avoided stigma. For example, some inaccurately location-named the virus and generated region- or culture-specific blame which in turn encouraged individual and community discrimination (Lou et al., 2022). In Ontario, Premier Ford referred to the ‘UK variant’ (CPAC, 2021a) and when COVID-19 cases were rising in a predominantly racialized region of Ontario, he stated, “I understand that a lot of cultures have massive weddings, bringing people from all over the world. You just can’t do it” (CPAC, 2021a). In fact, many of the people in that area were essential workers living in multigenerational homes, reliant on public transit, and without the luxury of working safely from home. Indeed, income, occupation, education, housing, and ethnicity all contributed to higher infection rates in racialized or lower-income areas across Canada (Nasser, 2020). By contrast, in Alberta, CMOH Hinshaw stated that communities experiencing rising case numbers were not to blame, and that additional actions were warranted to control the spread of COVID in those areas (CPAC, 2021d). Such statements place responsibility on governing bodies to assist and increase resources and help dispel the notion that vulnerable communities are responsible for insecure conditions.

Ultimately, politicians and public health officials largely failed to appreciate that a one-size communication approach does a disservice to diverse communities and the PHMs meant to protect them. While communication needs to be science-driven, messaging has to resonate with people. Values, knowledge, perspectives, and experiences must inform communication strategies or they risk being misunderstood as harsh or ill-conceived. This is not an easy balance; it demands infrastructure and ongoing, genuine community engagement with communities.

## Discussion

Subject to some exceptions, official communications did not meet the conditions of good communication; officials too rarely conveyed expectations and the best evidence supporting PHMs in a timely, clear, and consistent manner that was conscious of individual and community contexts. On transparency, PHMs were changed without clear evidence-based justifications, as demonstrated by the lifting of PHMs with case numbers still rising (Fig. 1). Releasing contradictory information and downplaying uncertainties validated suspicions held by vaccine-hesitant individuals (Bardosh et al., 2022). With respect to clarity, official communications were often ambiguous, with inconsistent and changing descriptions of PHMs. However, probably the most lacking and potentially damaging shortcoming revolved around engagement. In addition to being open, transparent, and honest about uncertainties (Hyland-Wood et al., 2021), communicators need to understand communities (Weible et al., 2020) and engage with and reflect community values, demonstrating why (and how) some values may need to be privileged over others in particular circumstances. Given that values can be understood differently, weighted differently, or result in differential acceptance of PHMs, failure to connect with communities at the level of values threatens the intentional dialogical encounter of communication, and undermines efforts to come to shared courses of action. Attention to the social and emotional context of information is important; communication that merges rational, emotional, and sensory elements in support of a consistent message builds trust and improves effectiveness (MacDonald et al., 2021; Hyland-Wood et al., 2021). However, value-cognizance was not often displayed, and communicators did not consistently know when to encourage individuals to consult (and locate) trusted healthcare providers for more detailed or nuanced information pertinent to the individual’s specific health and social circumstances.

The incoherence of the communication examined, and the infrequent reference to reliable evidence, has several important consequences. It impeded cross-jurisdictional comparison of PHMs, it undermined the identification and development of best public health practices across provinces, and it undermined public confidence and public trust, and ultimately compliance with PHMs (Lee & Li, 2021).

## Conclusion and recommendations

While there is clear need for further research into how Canadian governments might better communicate with Canadians during a public health emergency, our research supports several recommendations.

First, as demonstrated by the inconsistencies in terminology and consistently insufficient justifications for PHMs (and reference to evidence), the public health communication setting would benefit from clear and compelling federal guidance on best practices. Federal guidance identifying general best practice in meeting the conditions of good communication might better harmonize practices, allay confusion, ease social exhaustion, decrease hesitancy, and improve compliance. An obvious short-term objective should be to develop a shared or common lexicon to guide reporting and discourse across the country. This would facilitate consistent and accurate communication and limit unjustified jurisdiction-specific differences. The provincial experience also speaks to the need for timely federal communication adopting a national perspective during issues of national importance. This would take pressure off provincial/territorial and local officials.

Second, this (federally led) harmonization could probably only be achieved through improved collaboration between jurisdictions. Regular meetings between designated communicators at different levels of government to enhance consistency of messaging and facilitate the sharing of lessons learned would be beneficial, and again, federal authorities could (and should) take the lead in realizing this.

Third, governments across Canada must take steps to better recognize the critical importance of communities to good health outcomes. This demands improved platforms for decision-makers, communicators, and communities to engage. Supportive courses of action include communicator training (i.e., a program of communication training for public health officials addressing transparency, promptness, clarity, and engagement and compassion in their communication responsibilities) and better development of engagement infrastructure (i.e., processes by which communicators can ascertain and take into account community experience, collaborate with community members in decision-making and information dissemination, and involve local communicators sensitive to local needs and values).

Fourth, steps need to be taken to increase the diversity and representativeness of public officials and communicators. Ensuring that official communicators and decision-makers reflect Canada’s diversity should facilitate a retreat from one-size-fits-all communication strategies (Hyland-Wood et al., 2021) and move toward a more effective dialogical and context-sensitive approach.

### Limitations

This study benefitted from triangulation of multiple data sources that employed a combination of established methods: press releases and briefings, and interviews with public health experts, in light of changes in disease burden. Limitation of funding prevented a thorough analysis across all Canadian provincial and territorial jurisdictions that prevented complete national coverage, in part due to the pandemic pressures that target participants were under. In addition, for practical reasons, we relied on press briefings and associated material, and acknowledge that a variety of public health messaging avenues exist. Further research is underway on how different communities received and interpreted information during the pandemic, and what they specifically found to be effective and ineffective.

## Contributions to knowledge

What does this study add to existing knowledge?

- A comparative analysis of public health communication across 3 Canadian provinces during the COVID-19 pandemic that shows critical areas of failure.
- A framework for improved communication (transparency, promptness, clarity, value-sensitive engaged collaboration) and policy options to pursue same.

What are the key implications for public health interventions, practice, or policy?

- Identification of a framework for communication that emphasizes transparency, promptness, and clarity/consistency of communication, while remaining attentive to diverse community values through direct engagement with local actors, which public health communicators should be aware of.
- Communicator training which addresses best practice in relation to all four conditions of good communication.
- The public health communication and intervention environment would benefit from increased federal leadership, especially around lexicon, standards, and information exchange.
- Public health communication and governance would benefit from increased cross-jurisdictional collaboration, from government/official engagement with local communities, and from improved diversity of decision-makers and communicators.

## Data Availability

The study data are available from the corresponding author upon request.

## Appendices

**Table 1:** News release sources

Province	Source
Alberta	Government of Alberta. (2022). *News.* Alberta.ca. https://www.alberta.ca/news.aspx
Ontario	Government of Ontario. (2022). *Recent News.* Ontario Newsroom. https://news.ontario.ca/en
Nova Scotia	Government of Nova Scotia (2022). *News Releases.* https://novascotia.ca/news/

**Table 2:** Press briefing sources

Province	Date	Source
Alberta	March 15, 2020	Canadian Public Affairs Channel. (2020a, March 15). *Alberta COVID-19 Update: Continuing Care Facilities to Receive Extra Funding* https://www.cpac.ca/episode?id=bbad3cd1-2944-4621-b0f5-d3838b6dc053
Alberta	March 27, 2020	Canadian Public Affairs Channel. (2020b, March 27). *COVID-19: Alberta Orders Closure of Non-essential Businesses.* https://www.cpac.ca/episode?id=767daa1e-bbeb-4491-a036-36de9b2377f5
Alberta	April 7, 2020	Canadian Public Affairs Channel. (2020c, April 7). *Jason Kenney on COVID-19 Projections and Alberta’s Economy.* https://www.cpac.ca/episode?id=ec45050d-f882-47f1-ac6c-a31bf8d5b2c8
Ontario	April 11, 2020	Canadian Public Affairs Channel. (2020d, April 11). *Ontario Health Official Provides COVID-19 Update.* https://www.cpac.ca/episode?id=958103c6-af34-48aa-a78e-62a6b13c3373
Alberta	August 31, 2020	Canadian Public Affairs Channel. (2020e, August 31). *Alberta Update on COVID-19.* https://www.cpac.ca/episode?id=826df5f6-4455-4553-94df-9bd1145e2a21
Alberta	November 6, 2020	Canadian Public Affairs Channel. (2020f, November 6). *COVID-19: Alberta Expands Limits on Social Gatherings.* https://www.cpac.ca/episode?id=ca83b471-0707-4601-942d-c2ba636b31a7
Ontario	January 19, 2021	Canadian Public Affairs Channel. (2021a, January 19). *Ontario Premier Doug Ford on COVID-19 Vaccine Distribution.* https://www.cpac.ca/episode?id=3c71626b-c2d7-4efa-97c8-3c3aca304e54
Nova Scotia	March 4, 2021	Canadian Public Affairs Channel. (2021b, March 4). *Nova Scotia Premier Iain Rankin Comments After Cabinet Meeting.* https://www.cpac.ca/episode?id=464f5e89-96bd-49dd-b6af-66e6c2bd905d
Ontario	April 22, 2021	Canadian Public Affairs Channel. (2021c, April 22). *Ontario Premier Doug Ford Apologizes for Controversial COVID-19 Measures.* https://www.cpac.ca/episode?id=ae8a87a2-e328-4605-84b8-eafc7803b5df
Alberta	April 29, 2021	Canadian Public Affairs Channel. (2021d, April 29). *COVID-19: Alberta Implementing New Targeted Measures.* https://www.cpac.ca/episode?id=1dcf5eea-916a-4282-9c8a-99b465fdfaab
Alberta	May 5, 2021	Canadian Public Affairs Channel. (2021e, May 5). *Alberta Premier on Province*’*s New Restrictions, Vaccine Eligibility for Children*. https://www.cpac.ca/episode?id=4538757f-0e12-4fb7-9b3a-b620a7eb97de
Ontario	August 24, 2021	Canadian Public Affairs Channel. (2021f, August 24). *Ontario Health Official Provides COVID-19 Update.* https://www.cpac.ca/episode?id=d03dffc8-b69d-49ce-acf8-661c5fa7b26f
Nova Scotia	September 14, 2021	Canadian Public Affairs Channel. (2021g, September 14). *Nova Scotia Delays Move to Final Reopening Phase*. https://www.cpac.ca/episode?id=2301aadb-f3fd-4f5c-9ee8-189db4ad0c4e
Ontario	September 21, 2021	Canadian Public Affairs Channel. (2021h, September 21). *Ontario Health Official Provides COVID-19 Update.* https://www.cpac.ca/episode?id=43459550-ed81-4f46-b71d-2bb494a93a03
Nova Scotia	September 29, 2021	Canadian Public Affairs Channel. (2021i, September 29). *N.S. Update on Reopening Plan, New Vaccine Mandate.* https://www.cpac.ca/episode?id=f975fbfc-badc-413f-9844-6c9bc53355cb
